# Halo traction combined with posterior-only approach correction for cervical kyphosis with Neurofibromatosis-1: minimum 2 years follow-up

**DOI:** 10.1186/s12891-021-04864-8

**Published:** 2021-11-23

**Authors:** Hongqi Zhang, Ang Deng, Chaofeng Guo, Zhenhai Zhou, Lige Xiao

**Affiliations:** 1 Department of Spine Surgery and Orthopaedics, Xiangya Hospital, Central-South University, Changsha, China; 2 National Clinical Research Center for Geriatric Disorder, Xiangya Hospital, Central-South University, Changsha, China

**Keywords:** Neurofibromatosis-1, Cervical kyphosis, Halo traction, Posterior-only approach

## Abstract

**Background:**

Surgical management of cervical kyphosis in patients with NF-1 is a challenging task. Presently, anterior-only (AO), posterior-only (PO) and combined anterior-posterior (AP) spinal fusion are common surgical strategies. However, the choice of surgical strategy and application of Halo traction remain controversial. Few studies have shown and recommended posterior-only approach for cervical kyphosis correction in patients with NF-1. The aim of this study is to evaluate the safety and the effectiveness of halo Traction combined with posterior-only approach correction for treatment of cervical kyphosis with NF-1.

**Methods:**

Twenty-six patients with severe cervical kyphosis due to NF-1 were reviewed retrospectively between January 2010 and April 2018. All the cases underwent halo traction combined with posterior instrumentation and fusion surgery. Correction result, neurologic status and complications were analyzed.

**Results:**

In this study, cervical kyphosis Cobb angle decreased from initial 61.3 ± 19.7 degrees to postoperative 10.6 ± 3.7 degrees (P<0.01), with total correction rate of 82.7%, which consist of 45.8% from halo traction and 36.9% from surgical correction. JOA scores were improved from preoperative 13.3 ± 1.6 to postoperative 16.2 ± 0.7 (P<0.01). Neurological status was also improved. There was no correction loss and the neurological status was stable in mean 43 months follow-up. Three patients experienced minor complications and one patient underwent a second surgery.

**Conclusion:**

Halo traction combined with PO approach surgery is safe and effective method for cervical kyphosis correction in patients with NF-1. A satisfied correction result, and successful bone fusion can be achieved via this procedure, even improvement of neurological deficits can also be obtained. Our study suggested that halo traction combined with PO approach surgery is another consideration for cervical kyphosis correction in patients with NF-1.

## Background

Neurofibromatosis (NF) is an auto somal dominant hereditary disorder that consists of two subtypes: NF-1 and NF-2. NF-1 (von Recklinghausen’s disease) which is known as the most common form of neurofibromatosis with an incidence of 1 per 3000–4000 people worldwide, is associated with numerous clinical manifestations [1, 2]. Patients with NF-1 may present with a wide variety of clinical manifestations such as café-au-lait spots (over 90% affected), neurofibromas, Lisch nodules, and various skeletal abnormalities [3]. Spinal deformity is only seen in the subtype NF-1, and can be divided into two categories: dystrophic pattern and non-dystrophic pattern [4, 5].

Kyphosis is the most common deformity that occurs in the cervical spine of patients with NF-1 [6]. Although cervical kyphosis is asymptomatic in most patients with NF-1, it can also cause neck pain with occasional neurological complications like nerve root compromise and complete or incomplete spinal cord deficits, with induced life-threatening paralysis [4]. Some patients with the tendency to have progression of kyphosis and deterioration of neurological function were advised to accept early surgical intervention [7]. Surgical management depends on multiple factors such as patient age, kyphotic angle, flexibility, extent of vertebral dysplasia and neurological status. Presently, anterior-only (AO), posterior-only (PO) and combined anterior-posterior (AP) spinal fusion are common strategies for the management of cervical kyphosis in patients with NF-1 [8–10]. In recent years, single procedure and combined therapies have also been applied in the treatment of cervical kyphosis in patients with NF-1 [8, 9, 11], however, the choice of surgical strategy remains controversial.

In this study, we evaluated the safety and effectiveness of halo traction combined with posterior-only (PO) approach correction in the treatment of cervical kyphosis in patients with NF-1.

## Methods

### Clinical characteristics

Twenty-six patients with cervical kyphosis due to NF-1 treated in our institution were reviewed retrospectively between January 2010 and April 2018 (Table 1) including 11 males and 15 females, with average age of 16 years (range 7–29 years). Each patient presented with typical café-au-lait spots, and were definitely diagnosed with NF-1. Dystrophic changes were observed in 7 patients and 19 patients presented non-dystrophic. The involved cervical segments were from C2 to C7. In this study, 15 patients reflected cervical kyphosis while 11 patients presented additional neurological deficits such as neck pain, asthenia of limbs and positive pathological signs.

**Table 1:** Patients Demographic

Gender (M/F)	11/15
Mean age (yr)	16.8 ± 5.5
Max traction weight (kg)	3.9 ± 0.7
Period of traction (days)	23.2 ± 11.4
Period of follow-up (months)	43.0 ± 11.7
Involved vertebra	
C2-C5	4
C2-C6	4
C3-C5	4
C3-C6	8
C3-C7	4
C4-C6	1
C4-C7	1

### Inclusion and exclusion criteria

The inclusion criteria were as follows: (i) patients who definitive diagnosis of NF-1 and associated cervical kyphosis, cobb angle > 40 degrees, (ii) patients treat with halo traction combined with posterior-only approach correction, (iii) patients who underwent corrective surgery with a minimum of 2 years of follow-up. The exclusion criteria were as follows: (i) diagnosis of other types of neurofibromatosis, (ii) cervical kyphosis caused by congenital, traumatic, or idiopathic factors, (iii) a history of spine surgery.

### Imaging procedure

The deformity evaluation was performed on the anterior/posterior and lateral cervical radiographs. Dynamic lateral flexion and extension x-ray images and CT scans were also taken to evaluate the overall flexibility of the cervical spine. Magnetic resonance imaging (MRI) of the cervical spine was also obtained in all patients for further investigation of the intraspinal contents and compressive pathological features.

### Clinical evaluation

All patients in this study underwent halo traction combined with posterior fixation and fusion. The indications for surgical intervention in these patients were severe cervical kyphosis impairing movement, mechanical neck pain, different degrees of neurological deficit, or progression of cervical deformity. The neurological function evaluation was based on the JOA scores.

### Traction procedure

All patients underwent local anesthesia for halo placement. Patients were supine in the bed and halo traction applied in patients and started with a parallel traction. Then, a blanket roll was placed under the shoulders and the height was increased gradually. The traction direction was gradually changed from parallel traction to hyperextension traction. The initial traction weight was 2 kg, and it increased subsequently by 0.5 kg every 3 days reaching maximum traction efficiency. It can be implied that a maximum traction efficiency has been obtained because there was no improvement of kyphotic angle with increasing traction weight or a maximum traction weight that the patient can tolerate has reached (Generally, maximum traction weight is generally less than 1/6 of the patient’s weight.). Traction continued till there was no significant improvement in Cobb angle on weekly radiographic. Neurological examinations were performed 2 times per day, and if the patient had any complaint during the traction, the traction weight was reduced temporarily with the traction maintained.

### Surgical procedure

Posterior spinal fusion and correction were performed under general anesthesia. The operative procedure was performed under maintained maximum traction weight. The lateral mass screws and/or pedicle screws, hooks were placed at the levels of fixation via a middle incision. Generally, patients with osteoporosis and lower bone mineral density can cause a reduction of screw holding force. It is necessary to insert more mass and/or pedicle screws, hooks, to provide more anchor points, disperse correction force, and stabilize the correction result. It is also necessary to loosen facet joints and posterior column osteotomy (PCO) before correction, including SPOs and Ponte osteotomy. After posterior fixation and kyphosis correction, abundant bone graft (allograft bone and / or autogenous iliac bone) was performed to create conditions for posterior column fusion. Somatosensory evoked potentials (SEP), motor evoked potentials (MEP) and wake-up test were used to evaluate the spinal cord function during operative process. All the patients wore cervicothoracic orthosis for 3 months postoperatively.

### Evaluation methods

In order to evaluate the efficiency of halo traction combined with PO approach surgery for cervical kyphosis correction in patients with NF-1, correction result was measured with Cobb angle and following parameters were used.

Traction correction rate = (Pre-Tc Cobb-Post-Tc Cobb)/Pre-Tc Cobb× 100%.

Total correction rate = (Pre-Tc Cobb –Post-Op Cobb)/ Pre-Tc Cobb × 100%.

Surgical correction rate = Total correction rate – Traction correction rate.

(Pre-Tc = Pre-Traction, Post-Tc = Post-Traction, Post-Op = Postoperative)

In order to evaluate the safety of halo traction combined with PO approach surgery for cervical kyphosis correction in patients with NF-1, JOA scores were recorded to assess neurological functions.

### Statistical analysis

Data were managed and analyzed using SPSS 17.0. Data were presented with mean ± SD. A paired sample t-test was used to test for significant differences. A *P* value < 0.05 was considered statistically significant.

## Results

### Improvement of cobb angle

**Fig. 1:**
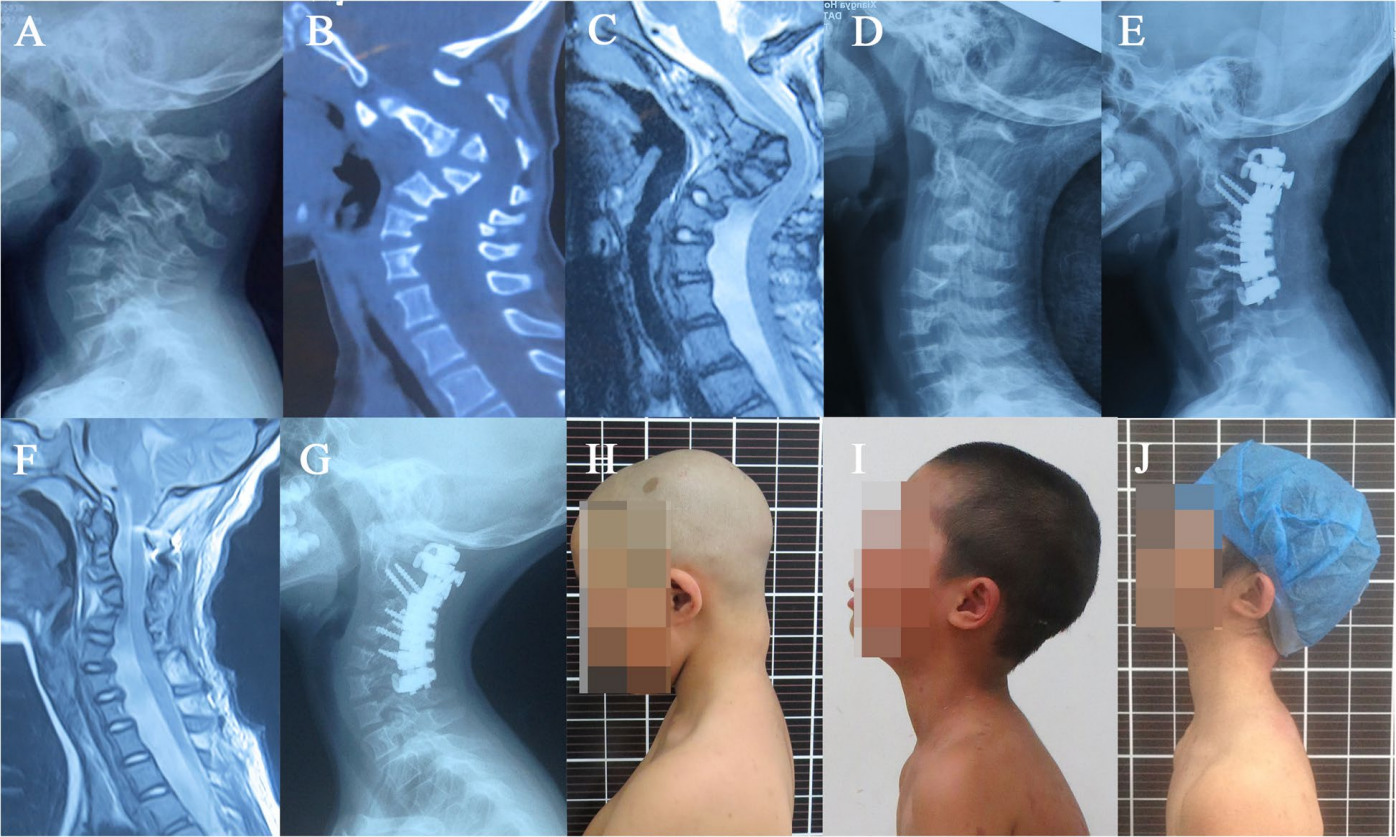
A 7-year-old case with NF-1 cervical kyphosis. Preoperative JOA score was 14. **A-C** Preoperative X-rays, CT showed severe kyphosis of 120° with severe dystrophic changes in cervical spine, and MRI demonstrated a mild compression from kyphosis. **D** After halo-traction, the kyphotic angle gradually improved from 120° to 52°. **E-F** Cercical kyphosis improved to 17° after surgery, and MRI showed the compression of spinal cord which was decompressed. **G** X-ray showed a solid bone union and no significant correction loss at follow-up. **H-J** Pre-op, post-op and latest follow-up clinical photos

**Fig. 2:**
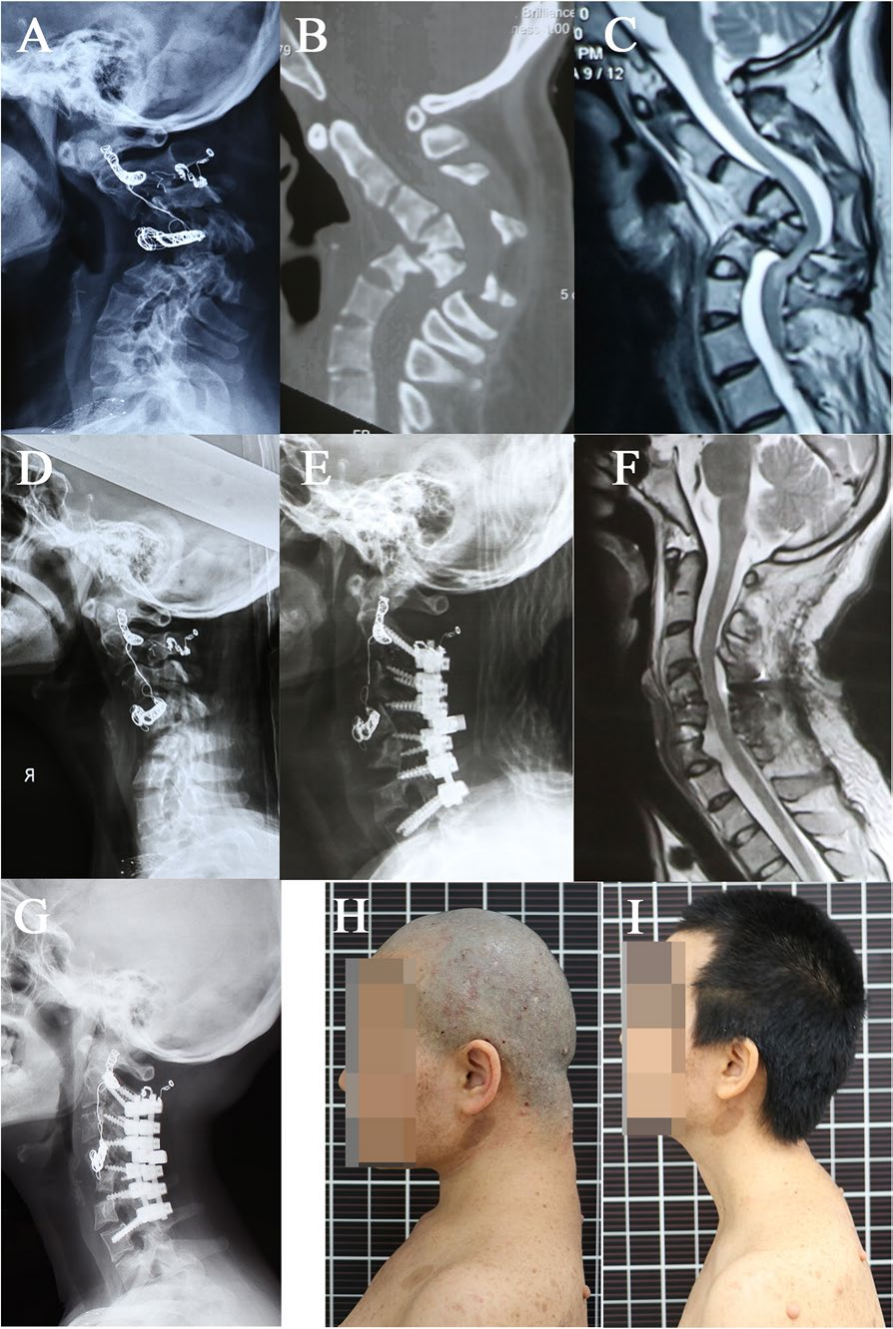
A 29-year-old case with NF-1 cervical kyphosis. Preoperative JOA score was 12. **A-C** Preoperative X-rays, CT showed severe kyphosis of 86° with severe dystrophic changes in cervical spine, and MRI demonstrated a mild compression from kyphosis. **D** After halo-traction, the kyphotic angle gradually improved from 86° to 45°. **E-F** Cercical kyphosis improved to 8° after surgery, and MRI showed the compression of spinal cord which was decompressed. **G** X-ray showed a solid bone union and no significant correction loss at follow-up. **H-I** Pre-op and latest follow-up clinical photos

All the patients underwent halo traction for 10–61 days. (mean 23.2 ± 11.4 days) with maximum weight of 5 kg (mean 3.9 ± 0.7 kg). Cobb angle decreased from Pre-Traction (Pre-Tc) 61.3 ± 19.7 degrees to Post-Traction (Post-Tc) 33.2 ± 11.2 degrees. *P* < 0.01. Traction correction rate was 45.8%. Postoperative Cobb angle was 10.6 ± 3.7 degrees. Surgical correction rate was 36.9%. All the patients were followed up for mean 43 months. Cobb angle was 9.7 ± 5.2 degrees in last follow up (typical case Figs. 1 and 2). There was no correction loss in follow-up. (Table 2).

**Table 2:** Improvement of kyphosis and statistical results

Parameters	Mean ± SD	*T* value	*P* value
Pre-Tc vs. Post-Tc	61.3 ± 19.7 vs. 33.2 ± 11.2	12.71	<0.01
Post-Tc vs. Post-Op	33.2 ± 11.2 vs. 10.6 ± 3.7	10.56	<0.01
Pre-Tc vs. Post-Op	61.3 ± 19.7 vs.10.6 ± 3.7	13.90	<0.01
Post-Op vs. Follow-up	10.6 ± 3.7 vs.9.7 ± 5.2	1.68	0.11

### Improvement of JOA scores

In this study, 15 patients presented only cervical kyphosis while 11 patients displayed additional neurological deficits. JOA scores improved from preoperative 13.3 ± 1.6 to postoperative 15.8 ± 0.9, P<0.01. JOA scores were 16.4 ± 0.6 in 2 years follow-up, the neurological status was stable in follow-up. (Table 3).

**Table 3:** Improvement of JOA score and statistical results

Parameters	Mean ± SD	*T* value	*P* value
Pre-Tc vs. Post-Op	13.3 ± 1.6 vs. 16.2 ± 0.7	−11.02	<0.01
Post-Op vs. Follow-up	16.2 ± 0.7 vs. 16.5 ± 0.6	−1.895	0.07

### Complications

In this study, 3 patients experienced halo pin loosing during traction. One patient underwent a second surgery because of unilateral upper limb paralysis, and the paralysis was fully recovered at 3 months follow-up. There were no systemic complications, pneumonia, thromboembolism, sepsis and peptic ulcers, which can arise due to prolonged bed rest.

## Discussion

Cervical abnormalities associated with NF-1 include enlarged neural foramina, cervical kyphosis, and gross cervical kyphosis with subluxation or dislocation. Kyphosis is the most common deformity and surgical management of this deformity has received little attention in literature reviews. For cervical kyphosis in NF-1, successful managements require early recognition, a more aggressive and reliable intervention to prevent disastrous worsening of the deformity. Several factors complicate the treatment and they are: i) a potential high risk of spinal cord injury during the correction. ii) difficulties in placing stable anchors in dystrophic vertebrae. iii) difficulty in obtaining solid bone fusion and iv) manipulation of the extreme degree of deformity in the presence of compromised cord may lead to severe cord damages and ischemia [7]. Three approaches were recommended to manage cervical kyphosis; Anterior-only (AO) approach, Posterior-only (PO) approach and combined anterior and posterior (AP) approach [10, 12]. Most previous studies have recommended AP approach, while a few studies have suggested anterior fusion or posterior fusion alone for cervical kyphosis associated with NF-1 because of fusion failure, pseudoarthrosis and correction loss in follow-up [7, 11]. A successful spinal fusion via single approach is fraught with difficulties in NF-1. Several scholars reported a high incidence of fusion failure which from 53 to 72% [13–16]. Moreover, a 23 and 7.5% incidence of fusion failure were observed in who managed by AP [16, 17]. ITherefore, how to manage the fusion failure is a challenge for kyphosis correction in NF-1. It is well known that NF-1 affects bone quality (osteoporosis) and quantity (vertebral body dysplasia) [18]. It is extremely difficult to place stable anchors where there are severe dystrophic changes in the cervical spine [19, 20]. Without stable anchors, the correction will eventually be suboptimal. Moreover, the other surgical challenge seen in NF-1 is lower bone mineral density (BMD). It has been reported that decreased bone BMD in both sexes at an early age is up to 50% of individuals with NF-1 [19]. Therefore, dystrophic and osteoporotic vertebral bodies may be insufficient to hold screws. Furthermore, preoperative halo traction can also have a negative impact on the BMD [21]. In this study, halo traction combined PO approach correction was used for the treatment of cervical kyphosis in patients with NF-1. In order to achieve stable fixation and successful bone fusion via PO approach, two points, more anchor points and abundant bone graft, in the operation are particularly important. More lateral mass and/or pedicle screws, hooks, even short screws, could provide more anchor points, which could disperse correction force and stabilize the correction result. Additionally, Abundant bone graft (allograft bone and / or autogenous iliac bone) was adopted to provide enough solid support for a successful bone fusion. As a result, the correction result was stable and bone fusion was successful in follow-up. After the operation, all the patients wore a cervicothoracic orthosis for 3 months, postoperative external fixation is mandatory to maintain the correction and to obtain solid bone union. Our study showed that with support of more anchors, abundant bone graft and postoperative external fixation, a stable fixation and successful bone fusion can be achieved via PO approach for cervical kyphosis correction in patients with NF-1.

The application of halo traction for treatment of cervical kyphosis in NF-1 is still controversial despite the use of halo spinal traction being widely used for treating severe spinal deformity [22–26]. Some scholars believed that traction provides a limited improvement of kyphosis [27, 28]. However, some authors believed that traction was very important adjuvant treatment for cervical kyphosis. Recent studies have further shown the safety and efficacy of halo traction as an operative adjunct procedure for cervical kyphosis correction.Halo traction could provide a slow and gradual correction while the patients were awake, which typically decreased the amount of corrective force that needed to be applied to the cervical spine. With the help of preoperative halo traction, partial correction of the deformity can be achieved to make the surgical procedure easier with less operative risk [21]. In this study, each patient underwent halo traction prior to the correction surgery. 45.8% traction correction rate was achieved eventually, and the cervical kyphosis was corrected to safe preoperative levels. Our study further proved that halo traction was safe and an effective adjuvant management for cervical kyphosis correction in NF-1.

Compared with several previous studies [7, 8, 10, 11, 29–32] (Table 4), halo traction combined PO approach surgery had a better correction. Halo traction provided the first level correction (45.8%), and surgery provided the second level correction (36.9%). Halo traction provided safe partial correction, and typically decreased the amount of corrective force that needed during the surgery. Some factors contribute to the high traction correction rate. In this group, most of patients were presented with flexible kyphosis without a rigid facet joint and they underwent prolonged traction (an average of 23.2 days, the maximum of 61 days). The soft tissue and facet joint can be released to the maximum as a result of long time and heavy traction. Additionally, there is no strong muscles and ligaments around cervical vertebrae and longtime hyperextension traction, which provided a continuous forward force in the cervical spine, and contributed to partial correction. Subsequently, intraoperative traction and PCO (posterior column osteotomy) was performed to provide a safe surgical correction rate and surgical correction rate eventually reached up to 82.7% via PO approach.

**Table 4:** Results of several previous studies

Authors	Year	Case	Pre-OP Cobb	Post-OP Cobb	Traction	Surgical procedure	Correction rate(%)
Yonezawa, I. et al [8]	2003	1	72	35	No	ASF + PSF	51.40
Laohacharoensombat, W. et al [29]	2010	1	120	55	Skull traction	ASF	54
J.M. Ma et al [30]	2011	8	58.5	2.5	No	540° comb procedure	95.7
F.L. Wu et al [11]	2012	1	125	30	Cervical suspensory traction	ASF + PSF	76
Kawabata, S. et al [7]	2013	3	140/81/72	50/15/27	Halo-gravity traction	ASF + PSF	64.3/81.5/62.5
Kevin R. Choksey et al [10]	2015	1	46	28	No	ASF	39.1
Y.F. Gu et al [32]	2018	7	67.7	12.4	Skull traction	ASF	83.1
J.C. Wang et al [31]	2019	10	82.0	27.3	Skull traction	ASF + PSF	66.7

The complication rate for the management of cervical kyphosis associated with NF-1 has not been well defined. It was considered that surgical correction of cervical kyphosis in patients with NF-1 has one of the highest rates of complications in cervical spine surgery [33]. Postsurgical complications included cutaneous infection, junctional kyphosis, kyphosis progression, fusion failure and pseudarthrosis at final follow-up. However, a previous study from Helenius [33] stated that the risk of complications did not differ significantly according to the surgical approach. Additionally, Preoperative halo traction was not associated with a lower risk of complications (44% compared with 69%, *p* = 0.24). In this study, the incidence rate of complications was kept in a relatively lower level. Only 3 patients experienced common traction related minor complications, and 1 patient occurred surgical related complications which was fully recovered after reoperation.

Improvement of neurological deficits via PO approach is a challenge in cervical kyphosis correction. NF-1 may present multiple levels of involvement and is likely to form complicated paraspinal and/or intraspinal tumors. A intraspinal tumors or tumors with nerve root invasion should be resected to achieve nerve decompression, and this procedure should be performed before a manipulation for deformity correction is done [34]. In addition, a progressive deformity can also lead to severe neurological impairment. When a neurological deficit is present in a young patient with NF-1, it is usually caused by increased kyphosis [35]. In this study, neurological deficits were improved to some degree via halo traction combined with PO approach correction. We inferred the following reasons to achieve the improvement of neurological deficits. Firstly, the neurological deficits caused by cervical kyphosis in NF-1 are mainly compression in front of the spine. It could be improved simultaneously with cervical kyphosis through a longtime standard traction. Secondly, the anterior column of the cervical spine is prolonged and the posterior column is relatively shortened via long time hyperextension traction. As a result, the compression in the front of spine can be improved. Thirdly, compression in cervical kyphosis is caused by apical vertebra and adjacent intervertebral disc. Traction can expand the intervertebral space and make intervertebral disc recovery to mean decompression. Also, the tolerance of spinal cord to ischemia and hypoxia was increased via a halo traction to reduce the risks of intra-operative neurological injury. Lastly, Posterior Column Osteotomy (Ponte osteotomy and SPOs) in apical region under intra-operative traction further released facet joint and shortened the posterior column, which decreased traction in the rear of spinal cord. Moreover, postoperative neurological deficit is another issue for cervical kyphosis correction in patients with NF-1. In a systematic literature review, Guzman et al. [36] reported that the prevalence of C5 nerve root palsy was 7.7% after anterior cervical procedures and 7.8% after posterior procedures; however, most of the deficits were resolved spontaneously during the 2-year follow-up period. In this study, postoperative neurological deficits occurred only in 1 patient who recovered after a revision surgery. We found that halo traction combined PO approach correction can also reduce the risk of postoperative neurological deficits.

There are some limitations that should be considered. One is the limited sample size of the included patients. The second limitation is that the medium- and long-term follow-up results should be further evaluated.

## Conclusion

This present study revealed that halo traction combined PO approach surgery is a safe and effective method for cervical kyphosis correction in patients with NF-1. Our data indicated that satisfied correction result and successful bone fusion can be achieved via this procedure, and improvement of neurological deficits can also be obtained. Our study suggested that halo traction combined PO approach surgery is another consideration for cervical kyphosis correction in patients with NF-1.

## Data Availability

The datasets analyzed during the current study are not publicly available but are available from the corresponding author on reasonable request.

## Abbreviations

NF: Neurofibromatosis
NF-1: Neurofibromatosis-1
AO: Anterior-only
PO: Posterior-only
AP: Combined anterior and posterior
CT: Computed tomography
MRI: Magnetic resonance imaging
JOA: Japanese Orthopaedic Association
PCO: Posterior Column Osteotomy
SEP: Somatosensory evoked potentials
MEP: Motor evoked potentials
Pre-Tc: Pre-Traction
Post-Tc: Post-Traction
Post-OP: Postoperative
BMD: Bone mineral density
